# Crosstalk between short- and long-term calorie restriction transcriptomic signatures with anxiety-like behavior, aging, and neurodegeneration: implications for drug repurposing

**DOI:** 10.3389/fnbeh.2023.1257881

**Published:** 2023-11-29

**Authors:** Agnes Hazi, Esmaeil Ebrahimie, Elizabeth A. Levay, Manijeh Mohammadi-Dehcheshmeh, Matt Zelko, Antonina Govic, Helen Nasser

**Affiliations:** ^1^School of Psychology and Public Health, La Trobe University, Melbourne, VIC, Australia; ^2^Genomics Research Platform, School of Agriculture, Biomedicine and Environment, La Trobe University, Melbourne, VIC, Australia; ^3^School of Animal and Veterinary Sciences, The University of Adelaide, Adelaide, SA, Australia; ^4^School of BioSciences, The University of Melbourne, Melbourne, VIC, Australia; ^5^Epigenes Australia Pty Ltd., Melbourne, VIC, Australia

**Keywords:** anxiety-like behavior, calorie restriction, energy uptake, aging, neurodegeneration, functional genomics, obesity

## Abstract

Calorie restriction (CR) is considered an effective intervention for anxiety, aging, and obesity. We investigated the effects of short- and long-term CR on behavior as well as transcriptome profiles in the hypothalamus, amygdala, prefrontal cortex, pituitary, and adrenal glands of Hooded Wistar and Long Evans male rats. A reduction in anxiety-like behavior, as assessed via the elevated plus maze, was observed in both short- and long-term CR. Despite this, short- and long-term CR regulated different sets of genes, leading to distinct transcriptomic signatures. The employed models were able to simultaneously analyze categorical and numerical variables, evaluating the effect of tissue type along with expression data. In all tissues, transcription factors, zinc finger protein 45-like and zinc finger BTB domain-containing two, were the top selected genes by the models in short and long-term CR treatments, respectively. Text mining identified associations between genes of the short-term CR signature and neurodegeneration, stress, and obesity and between genes of the long-term signature and the nervous system. Literature mining-based drug repurposing showed that alongside known CR mimetics such as resveratrol and rapamycin, candidates not typically associated with CR mimetics may be repurposed based on their interaction with transcriptomic signatures of CR. This study goes some way to unravelling the global effects of CR and opens new avenues for treatment for emotional disorders, neurodegeneration, and obesity.

## 1 Introduction

Calorie restriction (CR), defined as a reduction in *ad libitum* intake (by 10–40%) without malnutrition, is well-established to delay age-related diseases and concurrently augment longevity (Heilbronn and Ravussin, 2003). CR is also well documented to reduce age-related cognitive decline (Witte et al., 2009; Leclerc et al., 2020) and to exert improvements in emotional functioning, such as reducing anxiety-like behavior both in the short-term (Levay et al., 2007; Yamamoto et al., 2009; Kenny et al., 2014) and across the lifetime trajectory (Govic et al., 2022). Given the far-reaching implications of this intervention, CR has been the subject of a significant amount of research interest. Despite this, however, there is still an incomplete understanding of the cellular and molecular mechanisms mediating the anti-aging and anti-anxiety capacity of CR, and, correspondingly, no pharmacological intervention capable of reproducing the comprehensive effects of CR.

Recently, transcriptomic analysis of brain tissues involved in anxiety (hypothalamus, amygdala, and pituitary) revealed that long-term CR alters anxiety-promoting as well as neurodegeneration-associated genes (Govic et al., 2022). For example, we identified downregulated *C1QA* expression, a gene with documented links with anxiety-like behavior (Bai et al., 2022; Maras et al., 2022), ostensibly through its role in inflammation, and Alzheimer’s disease, through its role in synapse elimination and neuronal damage (Hong et al., 2016; Dejanovic et al., 2018; Wu et al., 2019). Moreover, attribute weighting algorithms identified *C1QA,* among others, as a tissue-independent signature of long-term CR. Crosstalk was also established between genes of the identified transcriptomic signature and anxiety, aging, and neurodegeneration through literature mining, underscoring *C1QA* as a potential biomarker of CR. This study exemplified the capacity of bioinformatic tools to analyze and interpret large-scale datasets and integrate such datasets with existing biological literature, thereby facilitating the identification of therapeutic targets for emotional disorders and neurodegeneration.

We are, however, at the beginning of understanding the impact of CR on cell systems biology. Many questions remain at the transcriptome level, for example: (1) Do responding genes and pathways to short-and long-term CR differ? (2) Which regulatory genes and non-coding RNAs are involved in the CR response? (3) Is it possible to achieve a universal tissue-independent transcriptomic signature of short-and long-term CR? (4) Are there drug candidates capable of mimicking the transcriptomic signature of CR?

Here, we utilized attribute weighting models to establish the transcriptomic signature of short- and long-term CR on a range of tissues involved in anxiety (hypothalamus, amygdala, pituitary, prefrontal cortex, and adrenal glands). The hypothalamus, pituitary, and adrenal glands are involved in the hypothalamic–pituitary–adrenal (HPA) axis, which is implicated in the neurogenesis of anxiety disorders (Tafet and Nemeroff, 2020). Moreover, the amygdala and the prefrontal cortex are key loci in the control of anxiety responses (Tye et al., 2011; Kenwood et al., 2022). Particular attention was paid to the response of transcription factors (TFs), receptors, transporters, chromatin-associated proteins, ligands, secretory proteins, and kinases to CR treatment, given their regulatory roles in gene expression and, in the case of transporters and ligands as they are key proteins for drug targets. We then adopted literature mining techniques, MedScan (Novichkova et al., 2003) implemented in the Pathway Studio webtool (Elsevier) (Nikitin et al., 2003), to establish crosstalk between responding genes in the transcriptomic signature of short- and long-term CR and anxiety. Since CR is widely recognized for its effects on aging and neurodegeneration (Flanagan et al., 2020; Fontana et al., 2021), these were also included as key terms in the literature mining analysis. Lastly, we further utilized literature mining to establish interactions between genes in the CR transcriptomic signatures and drug-repurposing candidates capable of mimicking the beneficial transcriptomic effects of CR.

## 2 Materials and methods

### 2.1 Animals

Adult male-specific pathogen-free Hooded Wistar (12- to 13- weeks old) and Long Evans (7–8 weeks old) rats were procured from Animal Resources Centre (Western Australia, Australia). Hooded Wistar rats were group housed (3–4 rats/cage) in large, open-top polypropylene basin cages (56.5 × 38.5 × 19.5 cm, l × w × h) while Long Evans rats were pair-housed in standard open-top plastic cages (38 × 27 × 15 cm, l × w × h). All rats were provided with standard rat chow (Barastoc, Ridley Corporation, VIC, Australia) and tap water *ad libitum*, wood shavings and shredded paper as bedding, and maintained under controlled temperature (23 ± 1°C) and lighting (reversed 12:12 h light: dark cycle; lights off at 1000 or 1,100 h) conditions. All procedures were conducted in accordance with the National Health and Medical Research Council of Australia Code of Practice for the Care of Experimental Animals and received by the RMIT University Animal Ethics Committee (approval number 1402) and La Trobe University Ethics Committee (approval number 18–15).

### 2.2 Short- and long-term calorie restriction treatments

Following a 2- to 3-week acclimation period, rats of each strain were randomly allocated into two treatment groups (short-term used Long Evans *n* = 14/group; long-term used Hooded Wistar *n* = 7–8/group): control and calorie restriction (CR). Controls were allowed *ad libitum* access to food throughout experimentation. The CR groups received 75% (25% restriction) of the amount of food consumed by the age- and strain-matched control rats, delivered daily within the hour before lights out (1000–1,100 h). Food intake of the CR group was initially determined by calculating the food intake of all rats across the last 48-h period of acclimation, and thereafter over 48-h every month. Food consumption of the control groups of both strains was consistent throughout experimentation with daily consumption ranging from 20 to 25 g per rat, equating to a mean daily food intake ranging from 15 to 19 g per rat for the CR groups. Long Evans rats underwent short-term CR, with CR extending for a total of 2.5 months, while the Hooded Wistar rats underwent long-term CR, with CR extending for 15 months. CR was initiated at approximately 3 months of age for both short-term and long-term CR rats. See Supplementary material S1 for body weight data across the duration of the experiments.

### 2.3 Behavioral testing

In the long-term CR experiment, behavioral testing in the elevated plus maze (EPM) and open field (OF) test occurred at 6, 12, and 18 months of age, corresponding to 4, 8, and 14 months of CR. These data were recently reported (Govic et al., 2022) and were used for comparison with the performed short-term CR in this study. Animals from the long-term CR study also underwent testing for acoustic startle reflex at 12 months of age. In the short-term CR experiment, behavioral testing in the EPM occurred 4 weeks following the initiation of CR. All animals were tested during the active dark portion of the light: dark cycle, approximately 1 h after lights-off and 2 h after the provision of food. To reduce the impact of circadian patterns on behavior, testing did not extend beyond the first half of the dark cycle and was preceded with a 30-min acclimation period to the testing conditions. Mazes/boxes were cleaned with 70% ethanol between tests. A closed-circuit camera mounted above the EPM allowed behavior to be recorded and tracked with Ethovision XT (Noldus, SDR Clinical Tech, Middle Cove, NSW, Australia) ethological tracking software which was operated in an adjacent room. While the individuals conducting the testing were not blind to group allocation, data collection and analysis via Ethovision were performed in a blind manner. Distance travelled (cm) was additionally calculated as an index of general locomotor behavior.

#### 2.3.1 Elevated plus maze

The EPM test was performed as described previously (Govic et al., 2022). Each rat was placed in the intersection (center) of the 50 cm elevated EPM apparatus facing an open arm (50 × 12 cm; l × w; 50 cm wall height for closed arms) and allowed 5 min of exploration. The distance rats travelled in the maze as well as the duration and frequency of entries into the open and closed arms was calculated by Ethovision XT, offline. Entry into each zone was operationally defined as having two paws in the zone. The ratio of open to total arm entries was calculated and presented here as an index of anxiety-like behavior.

#### 2.3.2 Statistical analysis of behavioral data

Four rats were excluded from the behavioral analysis (four control and one CR) for methodological reasons (rats either fell off the maze or demonstrated hypoactivity). Statistical analysis of behavior in the elevated plus maze for the remaining animals was conducted according to previously published workflows using the sequential effect existence and significance testing framework (Govic et al., 2022). As per this workflow, analysis of behavior was conducted using Bayesian generalized linear regression models using uninformative or weakly informative priors. Model diagnostics, including predictive checks and leave-one-out cross-validation, indicated model convergence and that diagnostic criteria were met. We include four requisite statistics to indicate the existence of an effect: Median estimate and 95% highest density interval (HDI) of the effect: *E_M_* (HDI lower bound, HDI upper bound), the probability of direction of the effect (*D_p_*), and proportion of the effect inside a region of practical equivalence (*ROPE_p_*).

Behavioral data are presented via box plots. Boxplots are a standardized graphical depiction of the distribution of a variable of interest. The interquartile range (IQR), representing the spread of 25–75% percentiles of the data, is displayed using the box. The whiskers extend 1.5 times the distance of the IQR below and above the box. Any data points outside these whiskers are deemed outliers and are plotted individually.

### 2.4 RNA preparation and sequencing

Following 2.5 months of CR (6 months of age) for the short-term CR group and 15 months of CR (at 18 months of age) for the long-term CR group, rats were humanely euthanized via an overdose of pentobarbital sodium (short-term study) or carbon dioxide asphyxiation (long-term study) and rapidly decapitated with the aid of a guillotine 2–4 h after lights-out. The hypothalamus, amygdala, pituitary, and prefrontal cortex (short-term CR only) were rapidly dissected out with the aid of a rat brain atlas and 1 mm coronal brain block (Braintree Scientific, Braintree, MA). Adrenal glands of the rats undergoing long-term CR were collected instead of the prefrontal cortex. Adrenal glands were collected from the short-term CR animals but due to methodological reasons were unable to be processed. Tissues were immediately flash frozen or stored in the Allprotect® Tissue Reagent (Qiagen, Hilden, Germany) and subsequently placed into a − 80°C freezer.

Total RNA was extracted from the rat brain tissue and adrenals (*n* = 5/region/group/strain) using the E.Z.N.A.® DNA/RNA Isolation Kit (Omega Bio-tek Inc., Georgia, USA) according to the manufacturer’s protocol. The nanodrop 2000c spectrophotometer (Thermo Scientific Inc., Waltham, MA, USA) was used to determine total RNA concentration, while integrity was assessed using the Agilent 2,200 TapeStation instrument (Agilent Technologies, Santa Clara, CA, USA). The TruSeq Stranded mRNA Library Prep Kit (Illumina, Inc., San Diego, CA, USA) was used to generate RNA sequencing libraries. The libraries were quantified and qualified using the High Sensitivity D1000 Screen Tape on an Agilent 2,200 TapeStation instrument. The libraries were normalized, pooled, and subjected to cluster and paired-end sequencing was performed for 150 cycles on a HiSeqX10 instrument (Illumina, Inc. San Diego, CA, USA) according to the manufacturer’s instructions.

### 2.5 Transcriptomic data analysis

The analysis of the generated sequencing reads was performed using CLC Genomics Workbench package 22 (QIAGEN) (Liu and Di, 2020) and Galaxy Australia^1^ (Jalili et al., 2020), including quality control of sequencing reads, trimming, mapping, and finding the differentially expressed genes. Rat reference genome and its annotation (*Rattus norvegicus*.mRatBN7.2) were downloaded from the Ensembl genome browser^2^ and used for mapping and expression analysis. Mapping was performed based on the following parameters: mismatch cost = 2, insertion cost = 3, deletion cost = 3, minimum length fraction = 0.8, and minimum similarity fraction = 0.8. The generalized linear model (GLM) based on negative binomial distribution (Robinson et al., 2010) was employed for differential expression analysis. The *p*-values were also corrected with false discovery rate (FDR) for multiple testing. The use of the GLM allows for curves to be fit to expression values without assuming that the error on the values is normally distributed. Fold changes were calculated from the GLM, which corrects for differences in library size between the samples and the effects of confounding factors. The Wald test was applied to calculate the *p*-values and FDR *p*-values for comparison of all group pairs. *p*-values and FDR *p*-values (corrected) were used for the selection of genes with significant differential expression in comparison of short-term CR against the control group and long-term CR against the control group in each of the studied brain regions.

### 2.6 Feature selection (attribute weighting) algorithms to find the responding genes to short- and long-term CR

The ensemble learning approach, based on voting of attribute weighting models, was employed in this study for biomarker discovery. The seven attribute weighting (feature selection) algorithms, including weighting by Info Gain, Info Gain Ratio, Rule, chi squared, Gini Index, Uncertainty, and Relief were applied, as previously described (Govic et al., 2022). Then, the genes and the studied tissue were ranked based on the overall received weights from attribute weighting models. The ones with higher cumulative weights were selected as key responsive genes. Attribute weighting models were successful in the discovery of tissue-independent transcriptomic signatures of CR.

PCA analysis based on the correlation matrix using transcriptomic signatures of each of the short-term and long-term CR treatments in each of the studied tissues was performed. The PCA analysis and PCA plot were generated by the Minitab21 statistical package.^3^ The signature was defined based on the top 20 responding genes to each condition derived overall weights of attribute weighting models.

### 2.7 Class-based transcriptomic analysis and literature mining

The top 20 genes selected by attribute weighting models as responding genes to short- and long-term CR were further classified based on protein classes, including protein kinase, protein phosphatase, receptor, RNA transcript, secretory protein, chromatin-associated protein, transcription factor, and transporter using gene ontology (GO) information, comparative GO, and the Pathway Studio tool (Ashburner et al., 2000; Fruzangohar et al., 2013; Consortium GO, 2019). All regulatory genes, secretory proteins, and extracellular biomarker genes that were identified in the top 20 were reported. We then employed literature mining by MedScan (Novichkova et al., 2003), a natural language processing (NLP) implemented in the Pathway Studio webtool (Elsevier) (Nikitin et al., 2003), to shed light on the relations between responding genes in the transcriptomic signature of short- and long-term CR and anxiety, depression, aging, and HPA axis from full texts of published articles, as previously described (Alanazi et al., 2018; Mohammadi-Dehcheshmeh et al., 2021; Govic et al., 2022). The confidence index was prefiltered by the software to note biological facts supported by >1 independent sentence(s) within the literature, with >3 being a high confidence, 2, medium confidence and 1, low confidence. Furthermore, the sentences detected by the software were manually checked to ensure the accuracy of the relation. In the field of literature/text mining, the robustness and confidence of a particular interaction is often assessed by examining the number of references, that correspond to the number of sentences in published resources, supporting that interaction. This approach has been applied by Farhadian et al. (2018) and Govic et al. (2022).

### 2.8 Drug repurposing and drug evaluation against the signature by text mining

Literature mining was applied to find the possible link between the available drugs and the responding genes to short- and long-term CR in this study. This signature was utilized to identify the optimal drug combination (drug repurposing) that mimics the effect of CR on the transcriptome.

## 3 Results

### 3.1 Short-term CR decreased anxiety-like behavior during exposure to the EPM

**Figure 1:**
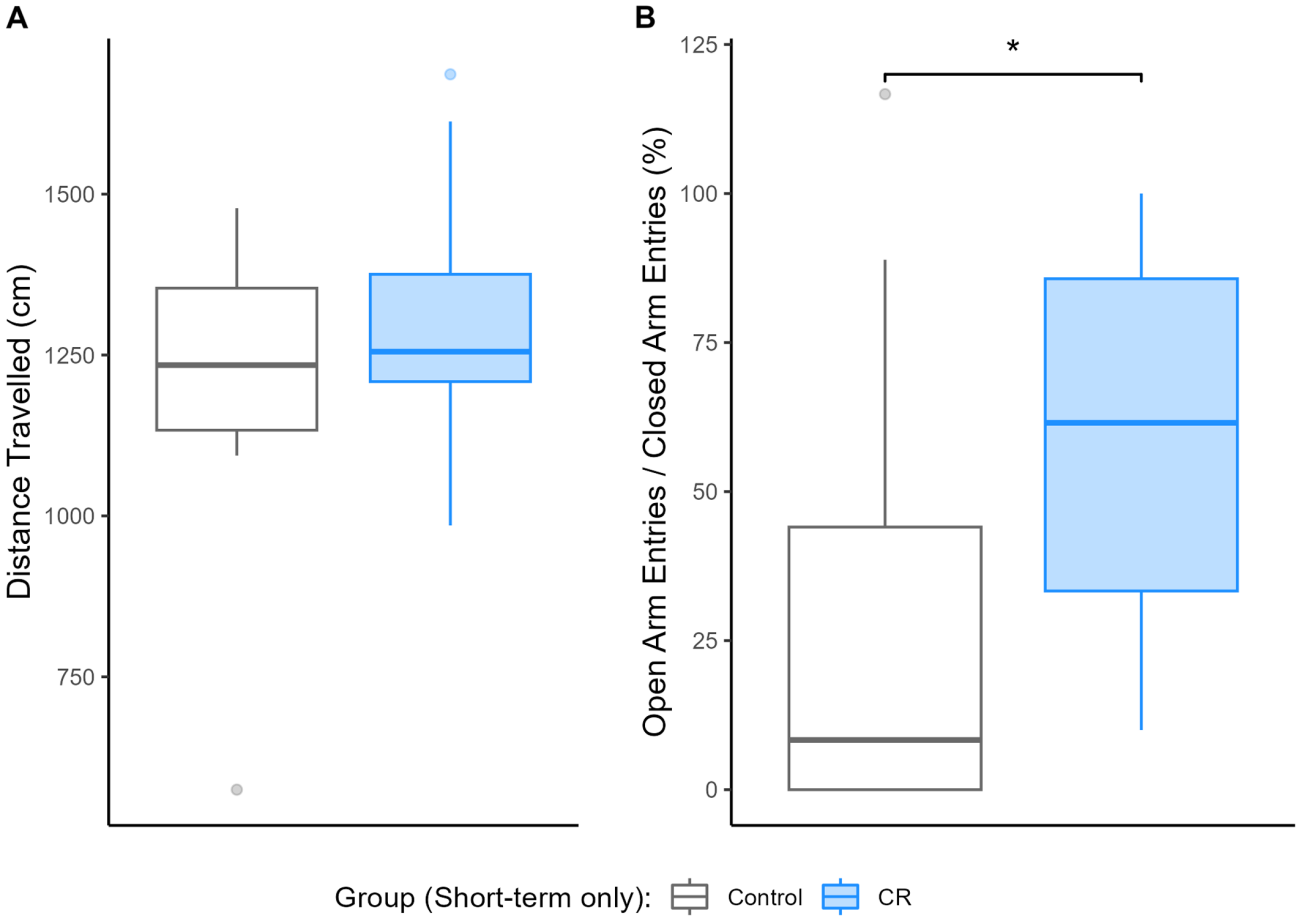
Impact of short-term calorie restriction (CR) (*n* = 13) relative to controls (*n* = 11) on anxiety-like behavior, based on performance on the elevated plus maze. Boxplots depicting **(A)** the distance travelled (cm) and **(B)** the ratio of open relative to closed arm entries (%) during testing. *Indicates practical equivalence is rejected.

As shown in Figure 1, short-term CR increased the proportional frequency of entries into the open arms relative to the closed arms [*E_M_ = 28.82, (−5.62, 60.44), D_p_ = 95%, ROPE_p_ < 0.01*] despite no difference in total distance travelled [*E_M_ = 12.13, (−223.5, 240.56), D_p_ = 54%, ROPE_p_ > 0.99*], indicating reduced anxiety-like behavior by short-term CR. Null hypothesis testing obtained the same result (Supplementary material S2). Anxiety-like behavior results for animals involved in the long-term experiment were recently published by Govic et al. (2022).

### 3.2 Comparing the transcriptome response between short- and long-term CR based on attribute weighting models

Supplementary material S3 provides the results of running seven attribute weighting models in ranking of the genes in response to short and long-term CR. Transcriptomic data for long-term CR animals has been previously published (Govic et al., 2022) but were here categorized by protein class (transcription factor, transporter, etc.) and compared to short-term CR data.

The overall (sum of the weights) ranks the genes in discriminating either short-term CR or long-term-CR from controls. The employed weighting models have the capability to analyze the effect of categorical variables of the tissue along with the numerical data of gene expression. The tissue received low overall weights in both short-term and long-term experiments demonstrating the possibility of the development of a tissue-independent signature.

**Figure 2:**
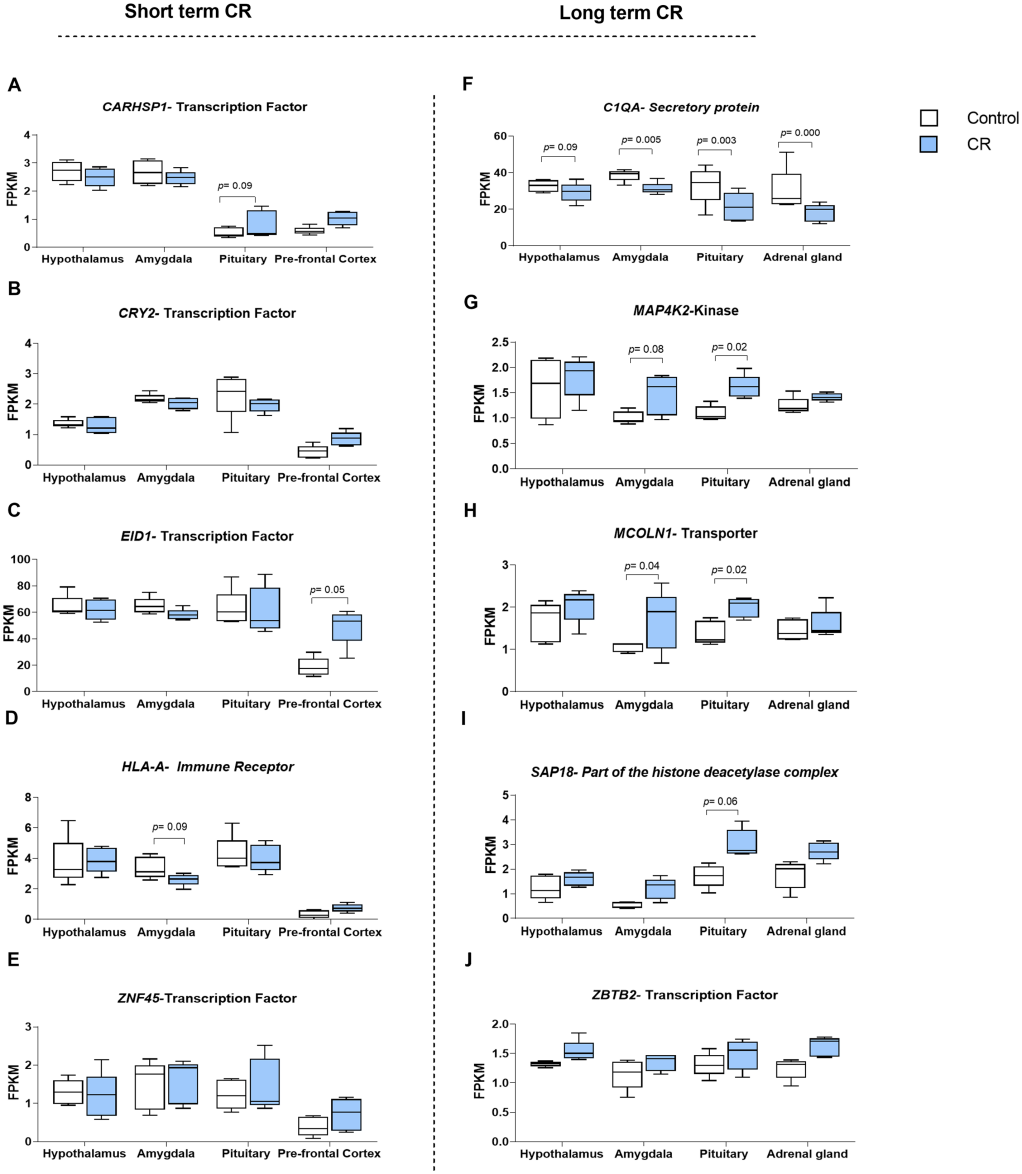
Genes belonging to the above mentioned protein classes were among the top 20 genes that received the highest overall weights from seven attribute weighting models employed for both short- **(A-E)** and long-term CR **(F-J)**. Part of the short term figures have been reproducedfrom Govic et al. (2022).

As it can be inferred from PCA analysis in Supplementary material S4, the developed short-term and long-term signatures, constructed from the top 20 responsive genes, were successful in distinguishing samples under short-term and long-term CR treatments from control samples. For example, in the prefrontal cortex, the short-term CR signature segregates CR samples from controls efficiently, accounting for 64.2% of data variation, respectively. In contrast, for long-term CR, PCA1 distinctly separated treated samples in both the amygdala and pituitary glands accounting for more than 68% of variation in the data. There is no similarity between the top 20 high-ranked genes between short and long-term CR (Supplementary material S3). It can therefore be concluded that there were ongoing transcriptomic changes with long-term CR beyond the short-term effects. The genes encoding key protein classes (transcription factors, ligands, receptors, kinases, secretory proteins, chromatin-associated proteins, and transporters) in studied tissues can be seen in Table 1 and Figure 2. Note that Figures 2F,G,J show reproductions from Govic et al. (2022) for purposes of comparison.

**Table 1:** Genes encoding the key protein classes, transcription factors, ligands, receptors, kinases, secretory proteins, chromatin-associated proteins, and transporters, that received high weights in response to short- and long-term calorie restriction (CR) in tissues involved in anxiety.

**CR type**	**Ensemble gene ID**	**Name**	**Class of** **protein**	**Weighting (feature selection) algorithm**							**Overall weight**	**Rank**
				**Info Gain Ratio**	**Rule**	**Chi squared**	**Gini Index**	**Uncertainty**	**Relief**	**Info Gain**		
Short	ENSRNOG00000055608	ZNF45	TF	1.0	1.0	0.9	1.0	0.8	0.4	1.0	6.1	1
Short	ENSRNOG00000007478	CRY2	TF	0.8	0.8	0.9	0.5	0.9	0.0	0.5	4.4	3
Short	ENSRNOG00000002610	CARHSP1	TF	0.8	1.0	0.5	0.7	0.6	0.1	0.7	4.3	4
Short	ENSRNOG00000008452	EID1	TF	0.5	1.0	0.7	0.5	0.8	0.2	0.4	4.1	13
Short	ENSRNOG00000030712	HLA-A	Immune receptor	0.5	1.0	1.0	0.2	1.0	0.2	0.1	4.0	15
												
Long	ENSRNOG00000019544	ZBTB2	TF	1.0	0.8	0.9	1.0	0.9	0.6	1.0	6.1	1
Long	ENSRNOG00000000975	MCOLN1	Transporter	0.6	0.6	1.0	0.6	0.9	0.8	0.6	5.1	6
Long	ENSRNOG00000021061	MAP4K2	Kinase	0.6	1.0	0.8	0.6	0.7	0.8	0.6	5.0	7
Long	ENSRNOG00000010732	SAP18	Part of the histone deacetylase complex	0.7	1.0	0.7	0.5	0.7	0.5	0.6	4.7	17
Long	ENSRNOG00000012807	C1QA	Secretory	0.6	0.9	0.8	0.6	0.7	0.5	0.5	4.6	20

The genes were ranked according to the weights that they received in discrimination of short- or long-term CR from controls. TF, transcription factor. Overall weight demonstrates the overall (sum) weight that a gene has received in discriminating CR samples from controls based on seven employed attribute weighting models. Rank demonstrates the rank of gene in response to CR according to the overall weight that was received in attribute weighting model.

Transcription factors *ZNF45*, *CRY2*, *EID1,* and *CARHSP1,* and the receptor *HLA-A* were key regulatory proteins identified in short-term CR tissues (hypothalamus, amygdala, pituitary, and prefrontal cortex). As can be inferred from Figure 2, the transcriptome response to long-term CR is more uniform than the response to short-term CR. All four regulatory proteins, *ZBTB2* (transcription factor), *MCOLN1* (transporter), *MAP4K2* (kinase), and *SAP18* (component of the histone deacetylase complex) were upregulated in response to long-term CR in the studied tissues (hypothalamus, amygdala, pituitary, and adrenal glands) as compared to the short-term genes, which were either up or downregulated depending on the tissue type. For long-term CR, in addition to these regulatory genes, *C1QA*, a secretory protein, was identified as being downregulated in all tissues except for the hypothalamus (which demonstrated a trend in this direction), as previously described (Govic et al., 2022).

### 3.3 Interaction of short CR-responding regulatory genes with neurodegeneration, anxiety, aging, and fertility

**Figure 3:**
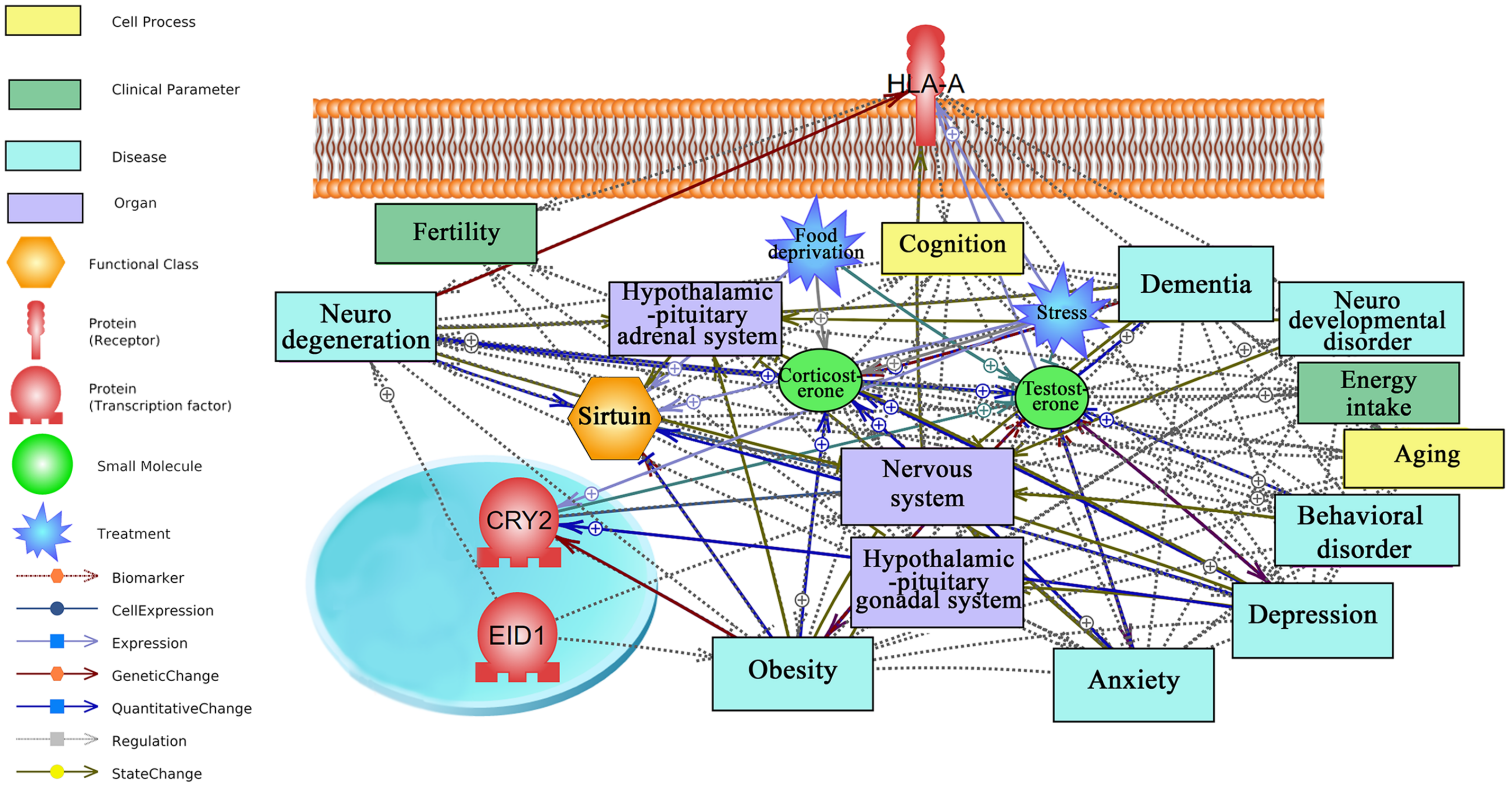
Text mining-derived network of top responding transcription factors and receptor, as identified by the attribute weighting analysis, to short-term CR with behavior (e.g., anxiety and depression), aging (e.g., neurodegeneration), regions/systems (e.g., HPA system), fertility, and stress. Detailed relationships, cellular locations, references, and mined sentences are provided in Supplementary material S5. ⨁ Denotes positive relation; ⊣ denotes inhibitory relation.

Based on literature mining, possible interactions of top responding transcription factors and receptors to short-term CR with neurodegeneration, anxiety, aging, and fertility are visualized in Figure 3. The underpinning mined sentences from literature and relationships are provided in Supplementary material S5. As mentioned above, the number of mined sentences in references that support a particular relationship is an index of confidence level where 3, 2, and 1 stand for high, medium, and low confidence, respectively.

*HLA-A* receptor, one of three genes encoding major histocompatibility complex class 1 (MHC1) and located on the cell membrane, is the hub gene in the regulatory network with many documented interactions with neurodegeneration, fertility, stress, and nervous system. While no interactions were observed with anxiety, *HLA-A* was found to have a positive regulatory role with aging, yielding a high confidence score (five mined sentences).

*CRY2*, a clock gene involved in circadian rhythms, is an interesting hub in the network that upregulates in response to depression, an affective disorder with high comorbidity with anxiety (Olfson et al., 2017). Interestingly, a negative regulatory role was demonstrated between *CRY2* and obesity as well as an association between mutations in *CRY2* and risk of developing obesity.

*EID1*, which represses transcription and regulates cell cycle and differentiation, plays a negative regulatory role in obesity and a positive regulatory role in neurodegeneration. Interestingly, there is no report on the top responding gene to short-term CR, *ZNF45* transcription factor, that opens a new avenue for further investigation and understanding of the regulatory mechanism of short-term CR at the transcriptomic level. Similarly, there were no interactions observed for *CARHSP1* in the network.

### 3.4 Interaction of long-term CR-responding regulatory and biomarker genes with neurodegeneration, anxiety, aging, and fertility

**Figure 4:**
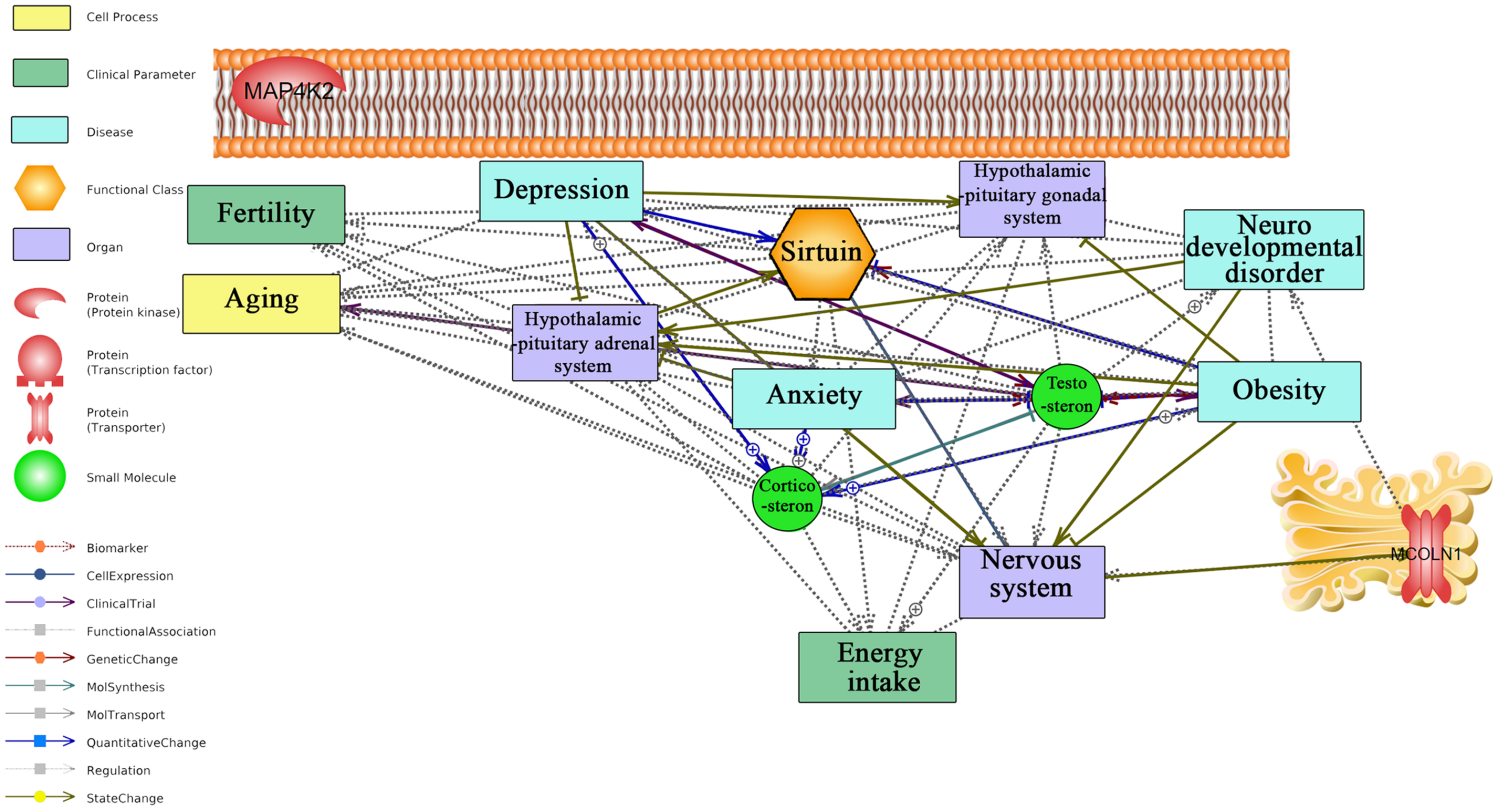
Text mining-derived network of top responding secretory protein, kinase, transporter, part of the histone deacetylase complex, and transcription factor, to long-term CR with behavior (e.g., anxiety and depression), aging, regions/systems (e.g., HPA system), and fertility. Detailed relationships, cellular locations, references, and mined sentences are provided in Supplementary material S6. ⨁ Denotes positive relation; ⊣ denotes inhibitory relation.

Figure 4 and Supplementary material S6 present the interaction of key classes of long-term responding genes as well as *C1QA* with anxiety, aging, and neurodegeneration. *C1QA* was identified as a hub in the regulatory network linking this gene with anxiety, depression, stress, aging, and neurodegeneration, as previously described (Govic et al., 2022). Our understanding of regulatory mechanisms of long-term CR is still in its infancy. *MCOLN1* receptor, located on Golgi, is the only regulatory long-term responding gene linked to neurodevelopmental disorder and the nervous system.

### 3.5 Drug repurposing

**Figure 5:**
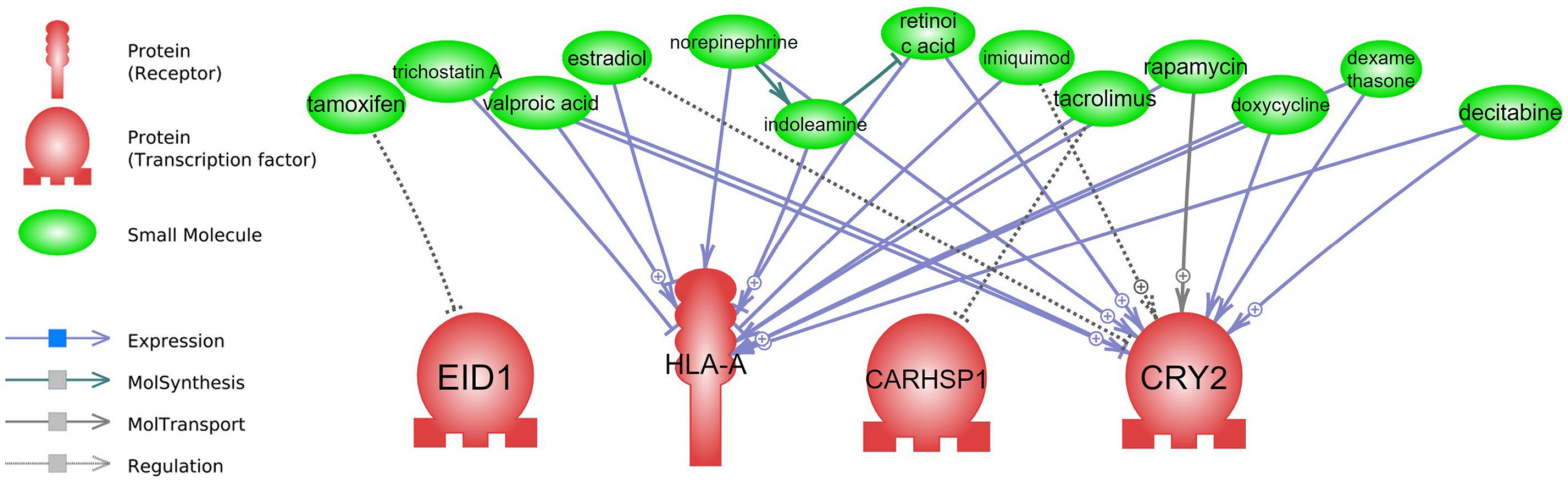
Drug repurposing: drugs that can interact with short-term CR transcriptomic signature. Detailed relationships, references, and mined sentences are provided in Supplementary material S7. ⨁ Denotes positive relation; ⊣ denotes inhibitory relation.

**Figure 6:**
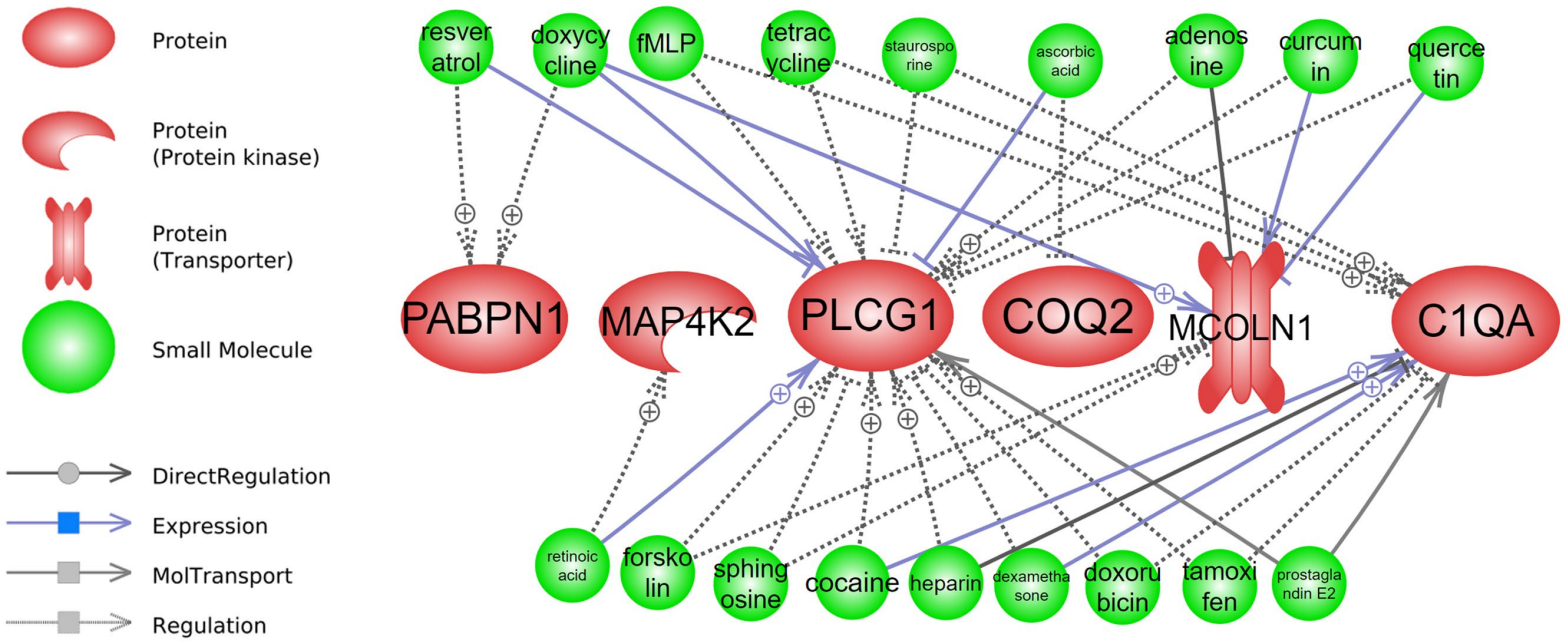
Drug repurposing: drugs that can interact with long-term CR transcriptomic signature. Detailed relationships, references, and mined sentences are provided in Supplementary material S8. ⨁ Denotes positive relation; ⊣ denotes inhibitory relation.

The results of drug repurposing in short and long-term CR are presented in Figures 5, 6 and Supplementary materials S7, S8, respectively.

For the short-term CR drug repurposing analysis, four of the regulatory genes from the attribute weighting analysis—*EID1*, *HLA-A, CARHSP1*, and *CRY2—*were identified as having interactions with known drugs (Figure 5 and Supplementary material S7). Only one drug was found to interact with *EID1* and *CARHSP1*, tamoxifen and tacrolimus, respectively, both demonstrating negative regulation. This was in contrast to *HLA-A* and *CRY2,* with multiple drugs showing interactions with these identified short-term CR genes. Some drugs, such as rapamycin, valproic acid, doxycycline, and dexamethasone, demonstrated various interactions (expression, positive/negative expression, and positive molecular transport) with *HLA-A* and *CRY2*. For example, valproic acid was negatively associated with the expression of *CRY2* (6 mined sentences, a high confidence score), where the same drug was positively associated with the expression of *HLA-A* (1 mined sentence, a low confidence score).

Regarding the long-term drug repurposing analysis, four of the top-responding genes from the attribute weighting analysis (*PABPN1*, *MAP4K2, PLCG*, and *COQ2*) as well as two regulatory genes identified in the class-based analysis (*MCOLN1* and *C1QA*) were found to have interactions with known drugs (Figure 6 and Supplementary material S8). Multiple and various interactions, such as positive/negative regulation and positive/negative expression, were demonstrated between known drugs and *PLCG1* and *C1QA*, while fewer interactions were demonstrated with *PABPN1*, *MAP4K2, COQ2,* and *MCOLN1*. Drugs such as doxycycline, resveratrol, tetracycline, dexamethasone, quercetin, and forskolin were found to interact with two to three of the long-term CR signature genes. For example, dexamethasone was positively related to the expression of *C1QA* (4 mined sentences and a high confidence score) and a negative regulatory relationship was identified between this drug and *PLCG1* (10 mined sentences and a high confidence score).

Interestingly, doxycycline and dexamethasone have interactions with genes in both short- and long-term CR (Figures 5, 6 and Supplementary materials S7, S8).

## 4 Discussion

Here, we exemplify the capacity of bioinformatic tools to integrate transcriptome-wide gene expression with comprehensive feature selection and literature mining to facilitate biomarker and drug repurposing candidate discovery. Attribute-weighing algorithms were employed to select a subset of regulatory genes as a tissue-independent transcriptomic signature of both short- and long-term CR. We then utilized literature mining to link these responsive genes in the short- and long-term transcriptomic signatures of CR with depression/anxiety, obesity, stress, aging, neurodegeneration, and the nervous system. Moreover, through literature mining, we identified drug repurposing candidates that may be capable of mimicking the CR signatures.

The behavioral results from the present study demonstrate that a short-term adult-onset CR of 25% results in an anxiolytic behavioral profile in male rats as evidenced by greater entries into the open arms of the EPM relative to the closed arms. This finding is congruous with the bulk of the research in this area demonstrating that short-term CR has an anxiolytic-like effect (Levay et al., 2007; Yamamoto et al., 2009; Kenny et al., 2014). We recently reported a comparable anxiolytic-like profile across the lifetime trajectory in the long-term CR animals from the current study (Govic et al., 2022).

To establish a tissue-independent transcriptomic signature of short- and long-term CR, we utilized seven attribute weighting algorithms, capable of mining numerical data of gene expression as well as categorical data of tissue type. Recently, the application of these algorithms has resulted in the development of a universal transcriptomic signature of long-term CR, independent from the categorical variable of tissue (with the levels of the hypothalamus, amygdala, pituitary, and adrenal glands) (Govic et al., 2022). Notably, in the current study, there was no overlap in the signatures identified for short- and long-term CR, potentially suggesting a differential transcriptomic outcome of cumulative CR relative to short-term CR. The lack of overlap may also reflect age-related changes in the transcriptome milieu, a commonly reported phenomenon (de Magalhaes et al., 2009; Palmer et al., 2021). It is essential to consider that the same behavioral phenotypic outcome, in this case, reduced anxiety-like behavior, can emerge from different molecular pathways. The distinct transcriptomic profiles we observed between short- and long-term CR suggest that the molecular mechanisms underlying their effects on behavior may differ. This, however, does not diminish the relevance of each signature but highlights the multifaceted nature of the biological response to CR. The outcomes of the feature selection (attribute weighting) models, partially illustrated in Figure 2, affirm the success of this study in identifying key responsive genes specific to both short-term and long-term CR treatments, irrespective of the tissue studied. While the PCA signatures effectively differentiate treated samples from the control ones, evidenced by significant percentages of the data’s variation accounted for by PCA1 in both short- and long-term CR. For instance, in the prefrontal cortex, the short-term CR signature segregates treated samples from controls efficiently. In contrast, for long-term CR, PCA1 distinctly separated treated samples in both the amygdala and pituitary glands. This differentiation has implications for how CR changes brain transcriptomic activity across time. It should also be noted that the long-term and short-term CR differed in more than the duration of CR, with strain, age at CR-onset, mode of euthanasia, and cage configuration differing between the studies. While the euthanasia method is unlikely to impact gene expression (Nakatsu et al., 2017), it is possible that the strain of rat, age at CR onset, and differing cage dimensions and number of animals per cage could have impacted the results.

Top responding genes within the signatures for short- and long-term CR were further classified based on protein classes (kinase, phosphatase, receptor, RNA transcript, transcription factor, secretory protein, chromatin-associated protein, and transporter) given the regulatory role of these in gene expression. We also identified secretory (extracellular) protein, *C1QA*, as a biomarker gene (Govic et al., 2022). Within the top 20 genes identified by attribute weighting algorithms, we identified five genes encoding regulatory proteins within both the short-term [*CARHSP1* (TF), *CRY2* (TF), *EID1* (TF), *HLA-A* (immune receptor), and *ZNF45* (TF)] and four in the long-term [*MAP4K2* (kinase), *MCOLN1* (transporter), *SAP18* (a component of the histone deacetylase complex), and *ZBTB2* (TF)] CR tissue. Zinc fingers, *ZNF45* and *ZBTB2*, received the highest weights in both short and long-term CR transcriptomic signatures, a notable finding given that zinc fingers not only play a role in transcriptional regulation but are also involved in signal transduction, DNA repair cell migration, and multiple other cellular processes (Cassandri et al., 2017).

For the short-term CR genes, literature mining-based network analysis identified *HLA-A*, found to be both downregulated and upregulated (depending on brain region) by CR, as a hub in the constructed network, linking this short-term CR responsive gene to aging and neurodegeneration, among others. *HLA-A* is one of three genes encoding major histocompatibility complex class 1 (MHC1), that is involved in the adaptive immune response as well as in brain development and plasticity (Elmer and McAllister, 2012). While no documented links were found between *HLA-A* and anxiety, links with aging revealed a robust positive relationship between *HLA-A* and aging (five mined sentences), where increased MHC1 expression in motoneurons is associated with aging (Edstrom et al., 2004) and increased MHC1 expression with the denervation of aging muscle (Tetruashvily et al., 2016). Literature mining also highlighted a relation between a genetic change in an *HLA-A* allele and Alzheimer’s disease (Wang et al., 2017).

*CRY2*, a clock gene involved in orchestrating circadian rhythms (Sjoholm et al., 2010), was found to be downregulated in all tissues, but the prefrontal cortex was also identified as a hub in the short-term CR regulatory network, relating this gene to obesity and the nervous system, with four mined sentences identified linking this gene with depression—a high confidence result. While literature mining-based network analysis did not identify any links with anxiety specifically, connections with depression were identified in Charrier et al. (2017), which reviews the involvement of clock genes in psychiatric disorders and includes a discussion of anxiety disorders. Specifically, *CRY1* and *CRY2* knockout mice have been demonstrated to display increased anxiety-related behavior (De Bundel et al., 2013). Relations between *CRY2* and depression suggest a positive association between the severity of depression and increased methylation of *CRY2* in women affected by overweight/obesity (Iodice et al., 2021). Additionally, a link between a *CRY2* locus and vulnerability to depression has also been demonstrated (Lavebratt et al., 2010). The results of these studies suggest that *CRY1* and *CRY2* may be important factors in mental health, particularly in anxiety-related behavior and depression vulnerability, but more research is needed to determine the potential diagnostic and therapeutic implications of these findings.

Regarding the literature mining-based network analysis for the long-term CR regulatory transcriptomic signature genes, *C1QA* was identified as a hub in the network with documented links to anxiety, depression, stress, aging, and neurodegeneration, as previously described (Govic et al., 2022). *MCOLN1* was found to have documented links to the nervous system and neurodevelopmental disorders, principally through the role of mutations to this gene in humans to a lysosomal storage disease, mucolipidosis type IV, a severe childhood neurodegenerative disease (Boudewyn and Walkley, 2019). No other relations were identified between long-term responding regulatory genes and search terms in the network analysis, underscoring the need for further research. Collectively, literature mining analysis suggests that long-term CR biomarker, *C1QA*, and regulatory gene *MCOLN1* may play important roles in several health conditions, including anxiety, depression, stress, and neurodegenerative diseases as well as aging itself. However, further research is needed to fully understand the clinical significance of these genes and their potential for therapeutic interventions. The identification of hub genes and their associated pathways may help to shed light on the mechanisms underlying the benefits of long-term CR and could be useful for the development of novel treatments for related disorders.

The distinct transcriptomic signatures observed between short-term CR and long-term CR carry significant implications for drug repurposing and drug discovery. The divergent transcriptomic profiles of short- and long-term CR necessitate the identification of unique drug candidates to emulate the beneficial effects associated with CR under both contexts. Considering this, we evaluated possible interactions between current drugs and the discovered transcriptomic signature of short- and long-term CR using literature mining, with the aim of identifying the best combination of drugs (drug repurposing) for the development of a CR mimetic. For the short-term signatures, multiple interactions were noted between *HLA-A* and *CRY2* and known drugs, such as rapamycin, valproic acid, doxycycline, and dexamethasone. Correspondingly, *PLCG1*, *MCOLN1*, and *C1QA*, long-term signature genes, demonstrated multiple interactions, including doxycycline, resveratrol, tetracycline, dexamethasone, quercetin, and forskolin. Regarding interactions with drugs recognized to mimic CR, rapamycin, a mTOR inhibitor and well-established anti-aging drug [see Selvarani et al., (2021) for a review] were flagged for the short-term signature genes *HLA-A* and *CRY2*. Similarly, the established CR mimetics resveratrol and quercetin (Hofer et al., 2021), both polyphenols found in fruits and vegetables, were flagged as interacting with long-term signatures *PLCG1* and *PABPN1* and *PLCG1* and *MCOLN1*, respectively. Notably, doxycycline, a second-generation antibiotic, and dexamethasone, a synthetic corticosteroid, have interactions with signature genes in both short- and long-term CR. Neither of these drugs has established CR mimetics; however, it is possible that, in combination with other drugs that interact with these genes, they may have some mimetic effects of CR. Further research would be needed to confirm this.

Regarding potential mimetics for affective disorders such as anxiety and depression specifically, literature mining analysis identified links with short-term CR signature gene *CRY2* and valproic acid, a histone deacetylase inhibitor utilized for manic episodes of bipolar disorder. Specifically, identified literature demonstrates that mood stabilizer valproic acid reduces *CRY2* expression in the amygdala (Ogden et al., 2004), suggesting the involvement of such clock genes in mood disorders (Liberman et al., 2018) and the potential to manipulate this pathway with CR mimetics. Overall, drug repurposing analyses identified several interactions between short- and long-term signature genes with well-established CR mimetics as well as highlighting some novel avenues for further exploration.

## 5 Conclusion

This is an innovative study that utilized bioinformatic techniques to unravel unanswered questions about the transcriptomic signature of short- and long-term CR. A class-based analysis of top responding genes in both the short- and long-term CR signatures identified five regulatory genes and four regulatory genes and an extracellular biomarker, respectively. Crosstalk between genes of these transcriptomic signatures with anxiety, depression, aging, and neurodegeneration was established by literature mining. Literature mining additionally established crosstalk between genes of the identified transcriptomic signature genes and drug repurposing candidates, identifying connections with established CR mimetics but also novel potential mimetics, such as doxycycline and dexamethasone. Collectively, we have successfully interrogated transcriptome-wide gene expression patterns in response to short- and long-term CR exploiting bioinformatic techniques so to contribute to the identification of therapeutic targets for emotional disorders, obesity, and neurodegeneration.

## Data availability statement

The datasets presented in this study can be found in online repositories. The names of the repository/repositories and accession number(s) can be found at: https://www.ebi.ac.uk/ena, PRJEB67383.

## Ethics statement

The animal study was approved by The Animal Ethics Committee of RMIT and La Trobe University Animal Ethics Committee. The study was conducted in accordance with the local legislation and institutional requirements.

## Author contributions

AH: Conceptualization, Funding acquisition, Investigation, Methodology, Project administration, Resources, Writing – original draft, Writing – review & editing. EE: Conceptualization, Data curation, Formal analysis, Methodology, Resources, Software, Visualization, Writing – original draft, Writing – review & editing. EL: Writing – original draft, Writing – review & editing. MM-D: Formal analysis, Visualization, Writing – original draft, Writing – review & editing. MZ: Formal analysis, Writing – original draft, Writing – review & editing. AG: Conceptualization, Formal analysis, Funding acquisition, Investigation, Methodology, Project administration, Resources, Supervision, Visualization, Writing – original draft, Writing – review & editing. HN: Conceptualization, Formal analysis, Funding acquisition, Investigation, Methodology, Resources, Visualization, Writing – original draft, Writing – review & editing.

## Footnotes

^1^https://usegalaxy.org.au/

^2^https://www.ensembl.org/index.html

^3^www.minitab.com
