# Supplementary material for: Crosstalk between short- and long-term calorie restriction transcriptomic signatures with anxiety-like behavior, aging, and neurodegeneration: implications for drug repurposing
Source: Front Behav Neurosci. 2023 Nov 29;17:1257881. doi: 10.3389/fnbeh.2023.1257881 (PMC10716537; doi:10.3389/fnbeh.2023.1257881)
Supplement: Supplementary file 1 [file Data_Sheet_1.zip › Supplementary material files/Supplementary material S2.DOCX]

## Supplementary 2.

## Short-term CR Decreased Anxiety-like Behavior during Exposure to the EPM

An independent samples t-test showed no significant difference in the distance travelled by control and CR rats, *t*(20) = -1.13, *p* = 0.271, *d* = -0.47, 95% CI [-1.28, 0.35].

A Shaprio-Wilk test indicated that the sample distribution was not normally distributed (*W* = 0.87, *p* < 0.01). Due to this violation, a Wilcoxon rank-sum test was conducted, which showed that normalised open-arm entries were significantly different between control and CR rats, *W* = 36, *p* = 0.041, *r* = 0.42.
