# Supplementary material for: Crosstalk between short- and long-term calorie restriction transcriptomic signatures with anxiety-like behavior, aging, and neurodegeneration: implications for drug repurposing
Source: Front Behav Neurosci. 2023 Nov 29;17:1257881. doi: 10.3389/fnbeh.2023.1257881 (PMC10716537; doi:10.3389/fnbeh.2023.1257881)

**Supplementary 4**.

PCA (Principal Component Analysis) analyses based on the transcriptomic signatures for both short-term and long-term calorie restriction treatments in each of the examined tissues. These signatures WERE constructed using the top 20 responsive genes determined through attribute weighting models in each of short-term and long-term calorie restriction conditions. These short-term and long-term signatures effectively differentiate samples subjected to calorie restriction from control samples.

**A.** PCA plot based on the 20 genes of short-term calorie restriction transcriptomic signature, distinguishing short-term calorie restriction (CR) samples from control ones in prefrontal cortex.


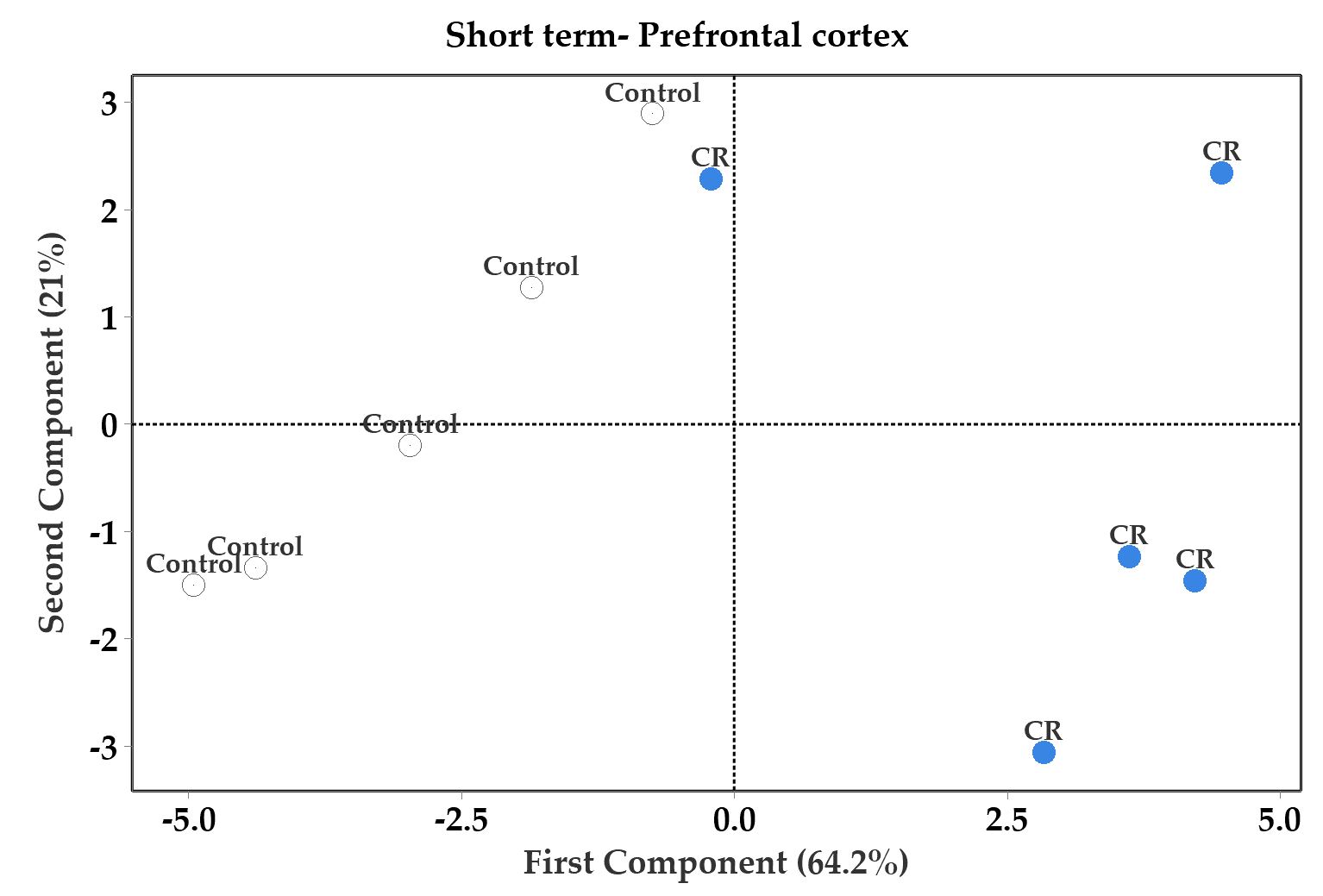


**B.** PCA plot based on the 20 genes of short-term calorie restriction transcriptomic signature, distinguishing short-term calorie restriction (CR) samples from control ones in amygdala.


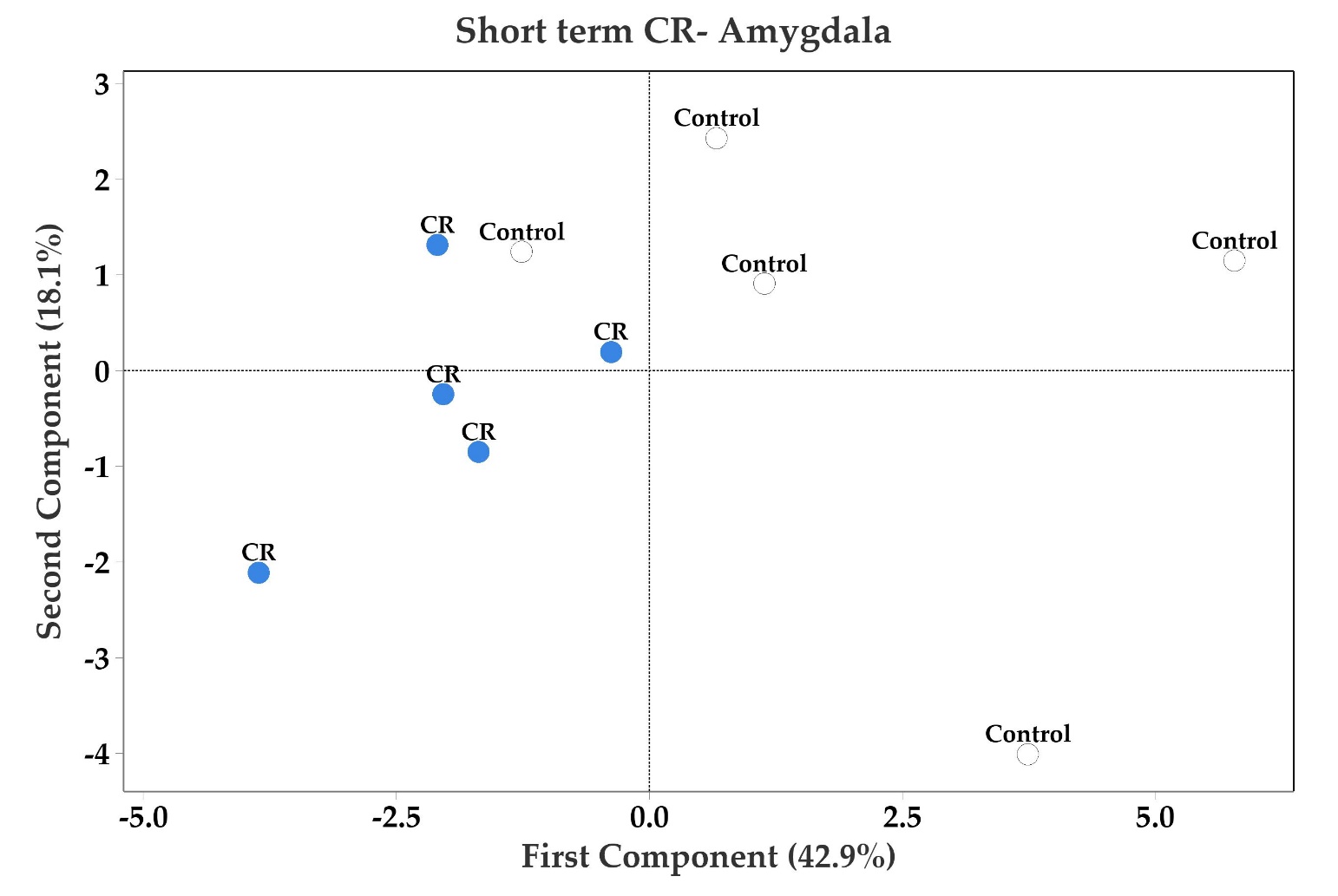


**C.** PCA plot based on the 20 genes of short-term calorie restriction transcriptomic signature, distinguishing short-term calorie restriction (CR) samples from control ones in hypothalamus.


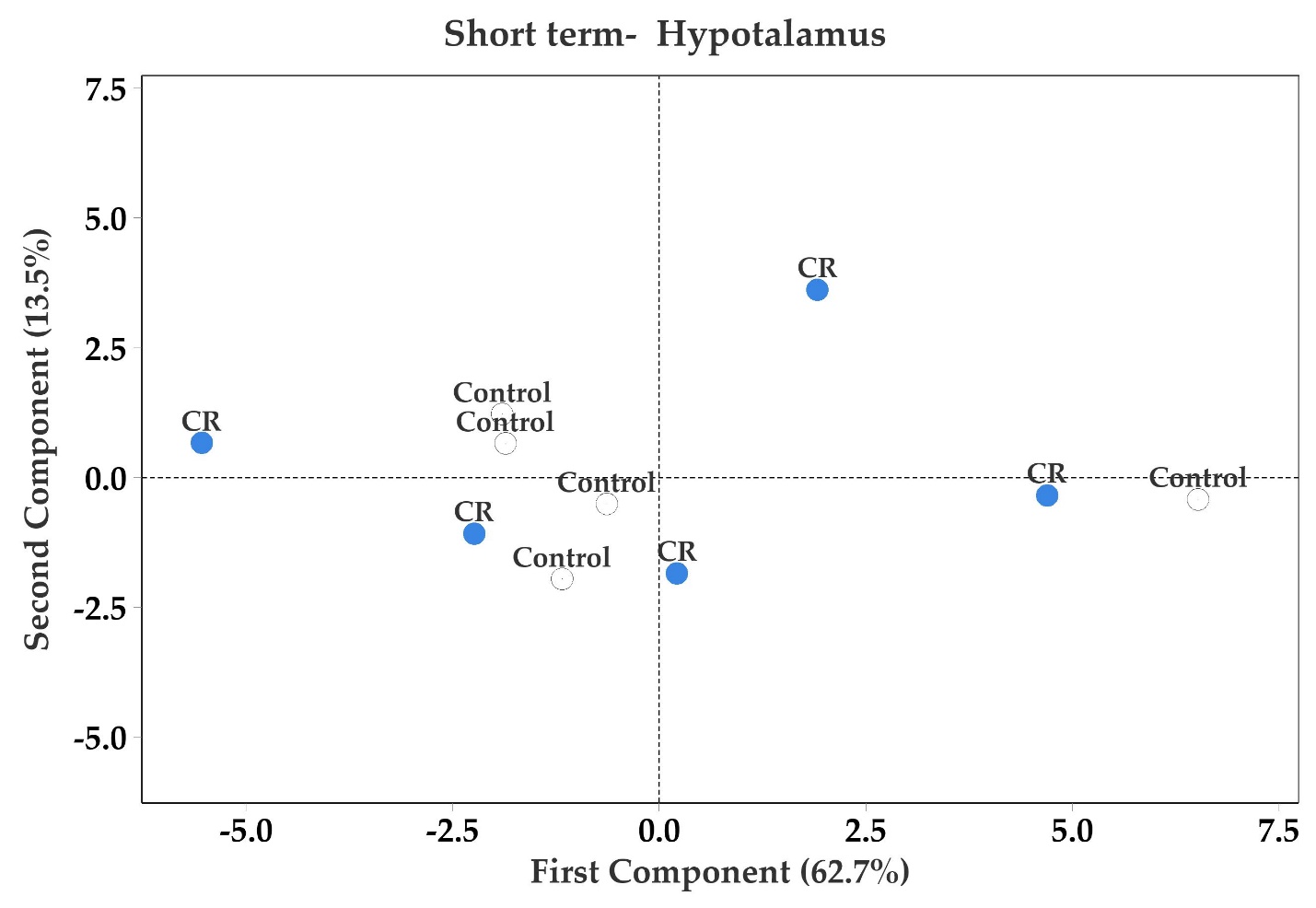


**D.** PCA plot based on the 20 genes of short-term calorie restriction transcriptomic signature, distinguishing short-term calorie restriction (CR) samples from control ones in pituitary.


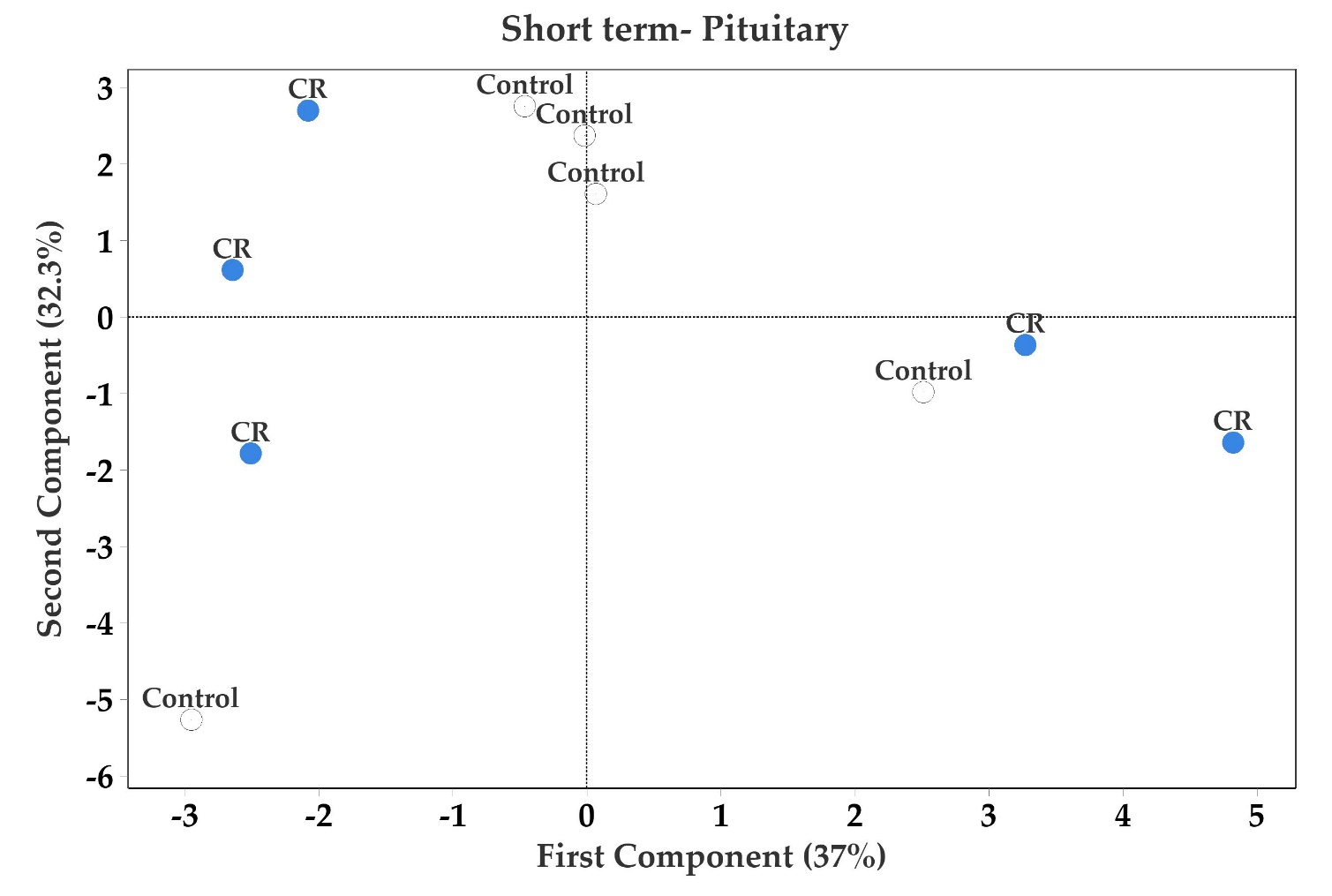


**E.** PCA plot based on the 20 genes of long-term calorie restriction transcriptomic signature, distinguishing long-term calorie restriction (CR) samples from control ones in pituitary.

**
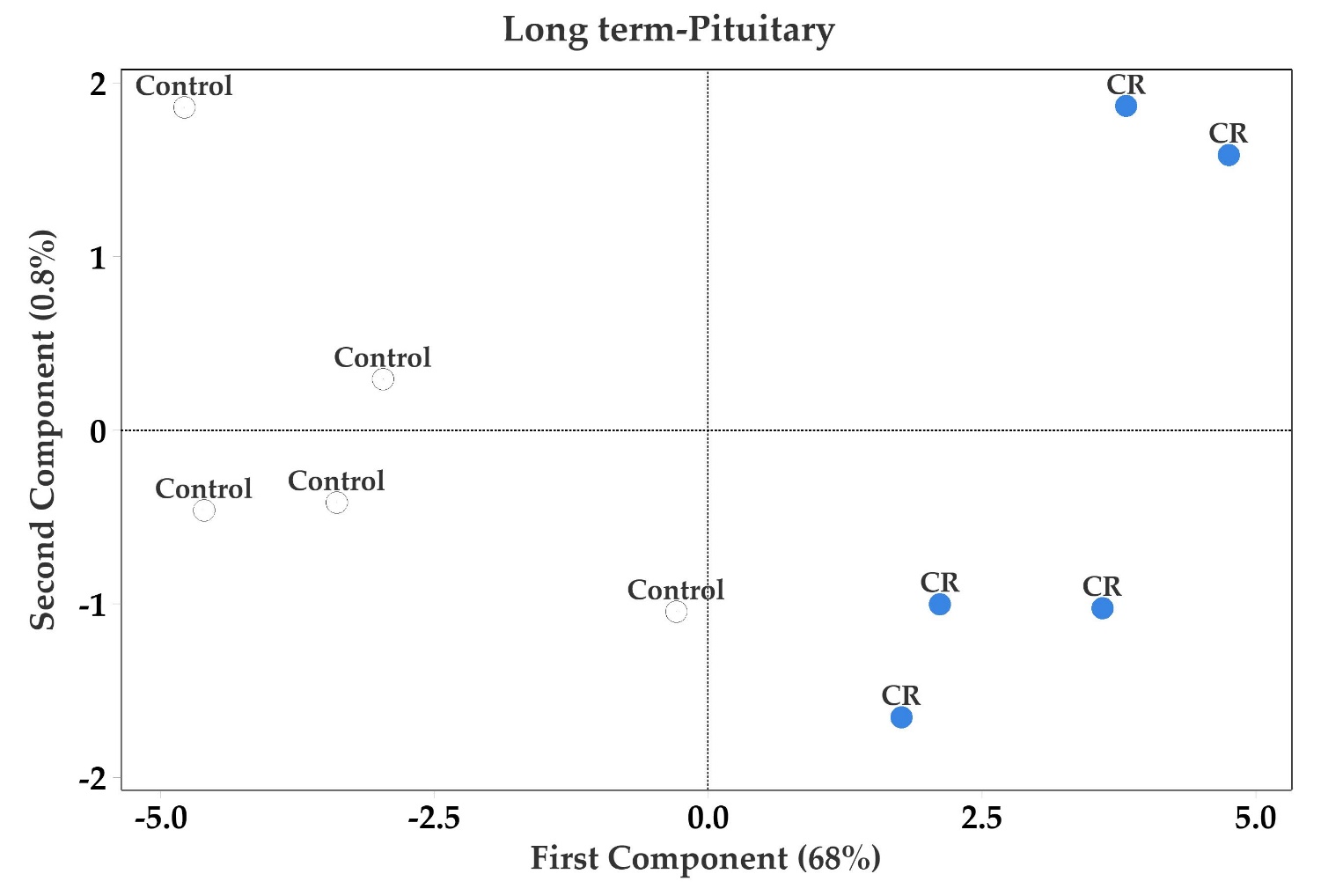
**

**F.** PCA plot based on the 20 genes of long-term calorie restriction transcriptomic signature, distinguishing long-term calorie restriction (CR) samples from control ones in adrenal glands.


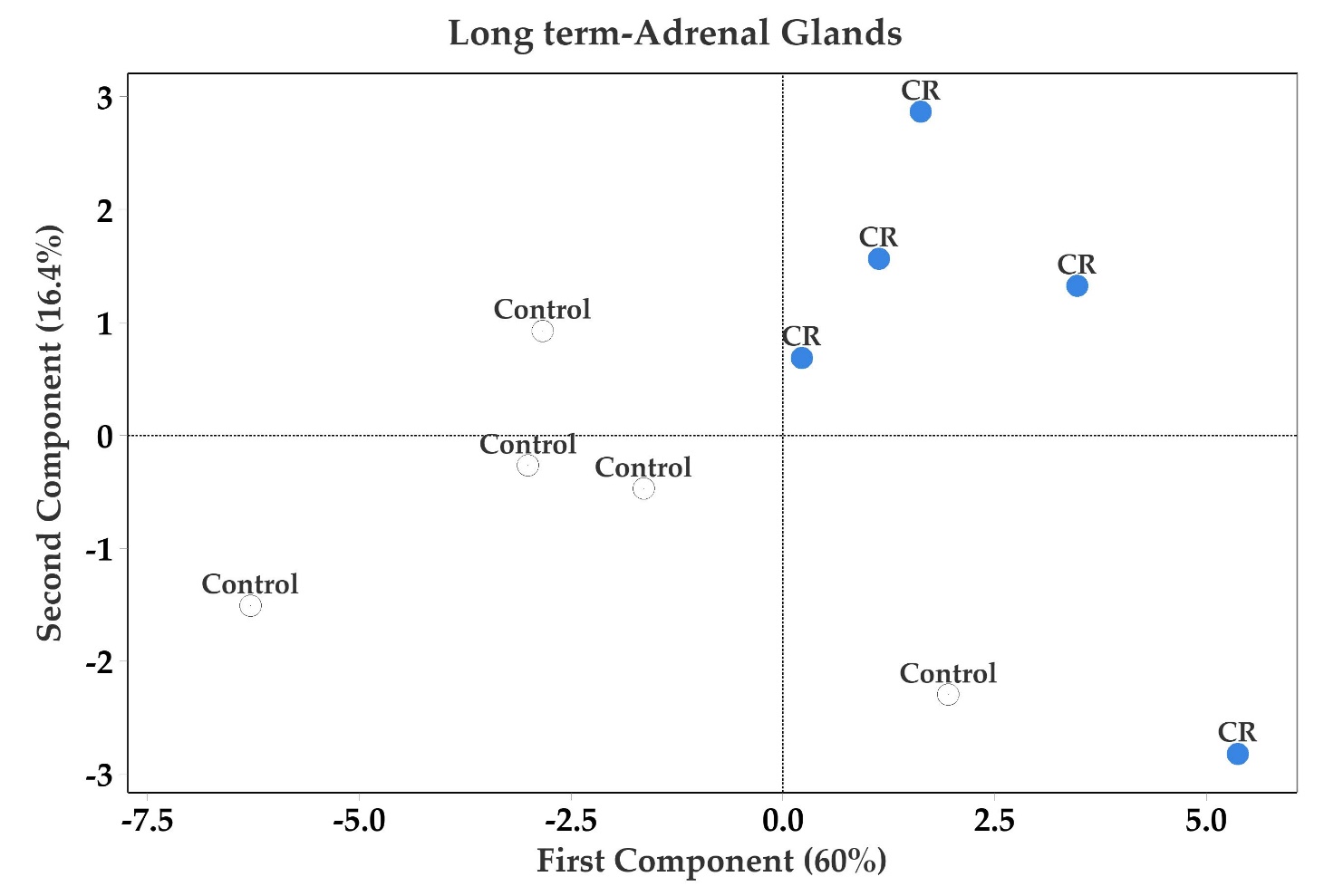


**G.** PCA plot based on the 20 genes of long-term calorie restriction transcriptomic signature, distinguishing long-term calorie restriction (CR) samples from control ones in amygdala.


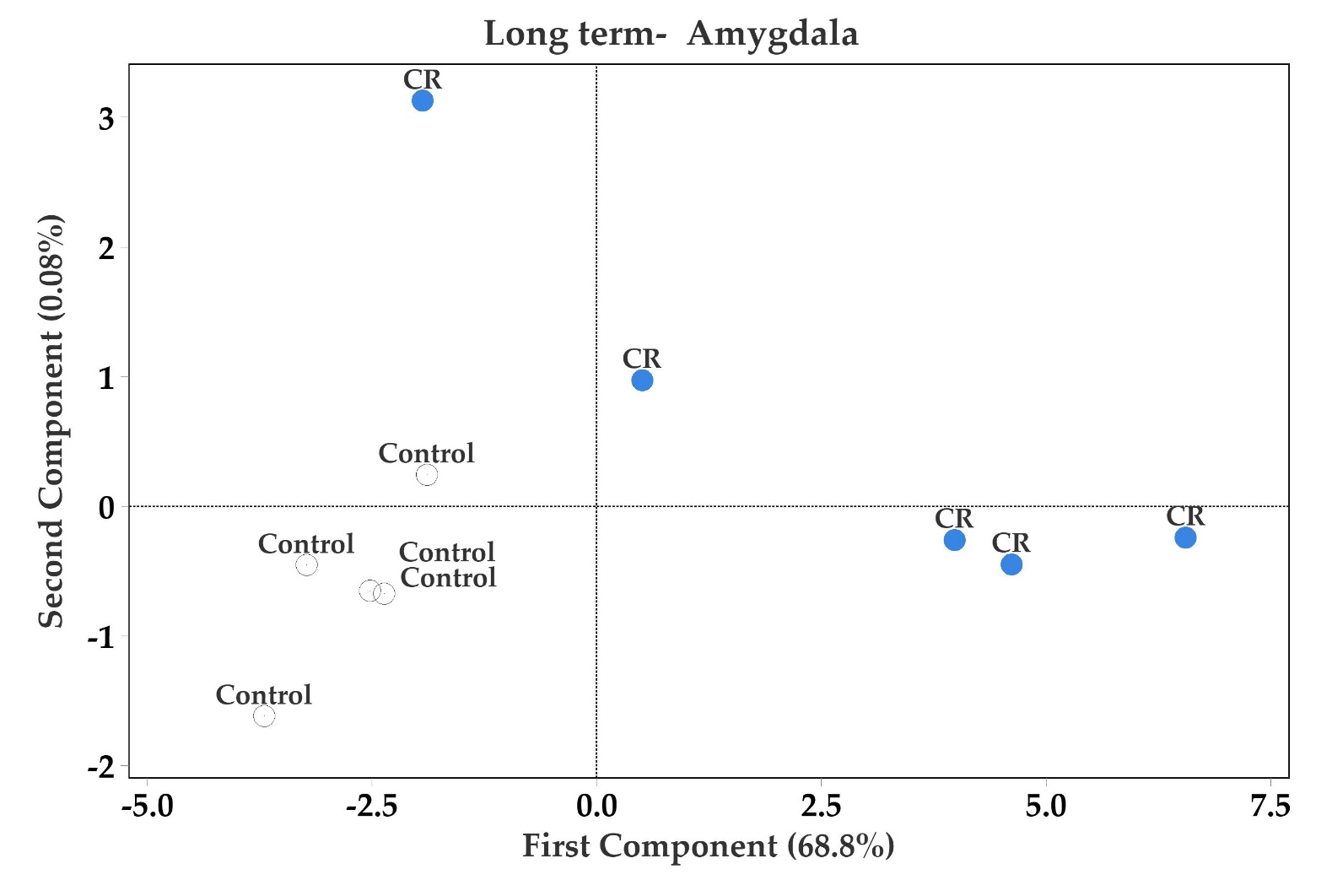


**H.** PCA plot based on the 20 genes of long-term calorie restriction transcriptomic signature, distinguishing long-term calorie restriction (CR) samples from control ones in hypothalamus.


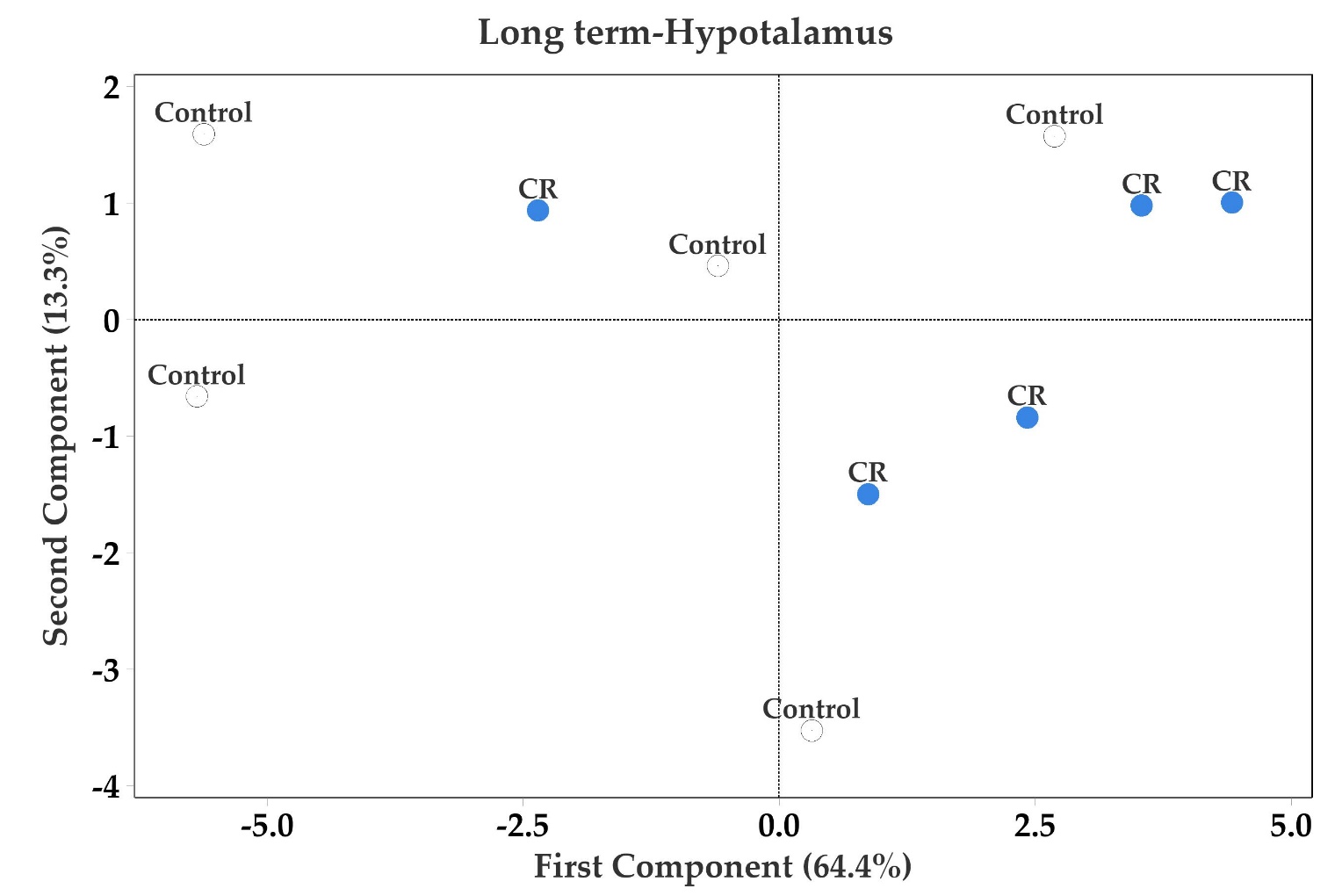

Supplement: Supplementary file 1 [file Data_Sheet_1.zip › Supplementary material files/Supplementary material S4.DOCX]
