# Supplementary figures and images for: Crosstalk between short- and long-term calorie restriction transcriptomic signatures with anxiety-like behavior, aging, and neurodegeneration: implications for drug repurposing
Source: Front Behav Neurosci. 2023 Nov 29;17:1257881. doi: 10.3389/fnbeh.2023.1257881 (PMC10716537; doi:10.3389/fnbeh.2023.1257881)

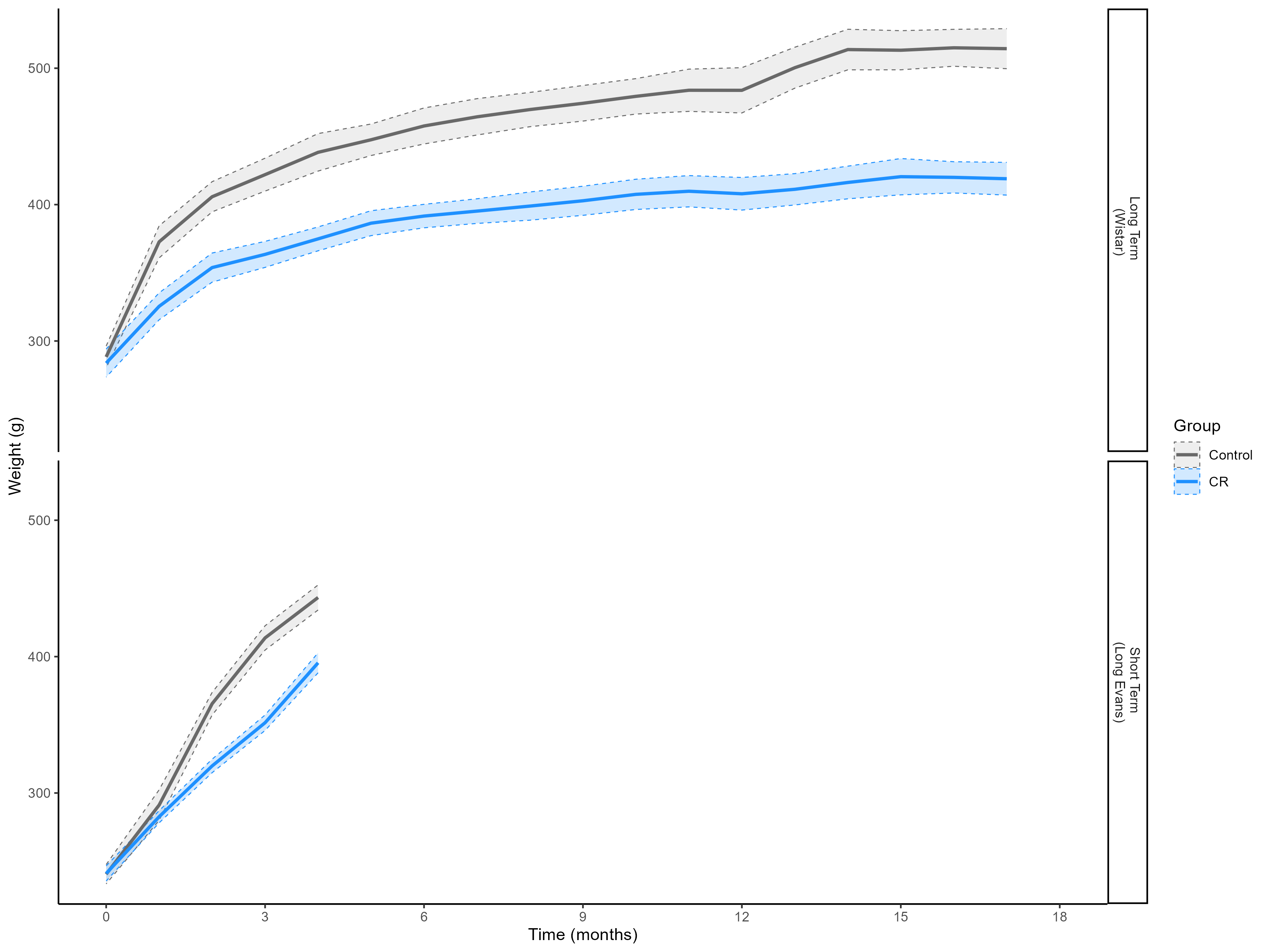

Supplement: Supplementary file 1 [file Data_Sheet_1.zip › Supplementary material files/Supplementary material S1.TIFF]
